# Sequential Washing with Electrolyzed Alkaline and Acidic Water Effectively Removes Pathogens from Metal Surfaces

**DOI:** 10.1371/journal.pone.0156058

**Published:** 2016-05-25

**Authors:** Yuichiro Nakano, Norihiko Akamatsu, Tsuyoshi Mori, Kazunori Sano, Katsuya Satoh, Takeshi Nagayasu, Yoshiaki Miyoshi, Tomomi Sugio, Hideyuki Sakai, Eiji Sakae, Kazuko Ichimiya, Masahisa Hamada, Takehisa Nakayama, Yuhzo Fujita, Katsunori Yanagihara, Noriyuki Nishida

**Affiliations:** 1 Department of Laboratory Medicine, Nagasaki University Graduate School of Biomedical Sciences, 1-7-1 Sakamoto, Nagasaki, Japan; 2 Department of Molecular Microbiology and Immunology, Nagasaki University Graduate School of Biomedical Sciences, 1-12-4 Sakamoto, Nagasaki, Japan; 3 Department of Physiology and Pharmacology, Faculty of Pharmaceutical Sciences, Fukuoka University, 8-19-1 Nanakuma, Jonan-ku, Fukuoka, Japan; 4 Department of Surgical Oncology, Nagasaki University Graduate School of Biomedical Sciences, 1-7-1 Sakamoto, Nagasaki, Japan; 5 Kyowakiden Industry Co., Ltd., 10–2 Kawaguchi, Nagasaki, Japan; 6 Kripton Co., Ltd., Dai 12 Daitetsu Bldg. 7F. 4-3-12 Yotsuya, Shinjuku-ku, Tokyo, Japan; 7 Teraoka Seikei Geka Hospital, 3-1-52 Minami-honjo, Fukuyama, Hiroshima, Japan; US Geological Survey, UNITED STATES

## Abstract

Removal of pathogenic organisms from reprocessed surgical instruments is essential to prevent iatrogenic infections. Some bacteria can make persistent biofilms on medical devices. Contamination of non-disposable equipment with prions also represents a serious risk to surgical patients. Efficient disinfection of prions from endoscopes and other instruments such as high-resolution cameras remains problematic because these instruments do not tolerate aggressive chemical or heat treatments. Herein, we develop a new washing system that uses both the alkaline and acidic water produced by electrolysis. Electrolyzed acidic water, containing HCl and HOCl as active substances, has been reported to be an effective disinfectant. A 0.15% NaCl solution was electrolyzed and used immediately to wash bio-contaminated stainless steel model systems with alkaline water (pH 11.9) with sonication, and then with acidic water (pH 2.7) without sonication. Two bacterial species (*Staphylococcus aureus* and *Pseudomonas aeruginosa*) and a fungus (*Candida albicans*) were effectively removed or inactivated by the washing process. In addition, this process effectively removed or inactivated prions from the stainless steel surfaces. This washing system will be potentially useful for the disinfection of clinical devices such as neuroendoscopes because electrolyzed water is gentle to both patients and equipment and is environmentally sound.

## Introduction

Performing surgical treatments under rigid endoscopy has been recommended and dramatically changes the approach to treating diseases because these interventions are less invasive than traditional methods and result in better overall outcomes [1, 2]. Thoracoscopic resection of esophageal and neuroendoscopic removal of pituitary tumors are recently developed and widely adopted examples of endoscopic surgeries [1, 3–5]. Some bacteria are capable of forming biofilms on the endoscope and cause iatrogenic infection [6, 7]. Usual decontamination procedures for endoscopes do not prevent iatrogenic transmission of prions, representing a serious problem in hospitals [8]. Endoscopes, and other instruments such as high-resolution cameras, are expensive and easily damaged by chemicals or heat because of their complex designs with multiple inner structures. A gentle disinfection process that is effective at removing or destroying pathogens is needed for these sorts of equipment [6, 9].

Electrolyzed acidic water is a relatively new disinfectant used for endoscope reprocessing [10, 11]. Electrolyzed acidic water eliminates bacteria and viruses from surgical instruments [12–14]. Electrolysis of a sodium chloride solution produces acidic water that has excellent biocidal activity and leaves no toxic residue that is harmful to patient tissues or irritating to the respiratory tract, eyes, and skin [11]. Moreover, alkaline water inactivates transmissible spongiform encephalopathies, the pathogens responsible for prion diseases—such as bovine spongiform encephalopathy in cattle, scrapie in sheep and sporadic, familial, iatrogenic, and variant forms of Creutzfeldt–Jakob disease (CJD) in humans [15, 16]. In this study, we develop a new washing procedure for rigid endoscopes using electrolyzed water and sonication. This washing procedure consists of treatment with alkaline water that aids the removal of protein contamination and acidic water that contains the biocidal agents HCl and HOCl [12–14, 17–19]. We evaluate the effects of sequential washing to remove or inactivate contamination by bacterial pathogens *Staphylococcus aureus* and *Pseudomonas aeruginosa*, the fungus *Candida albicans*, and human prions.

## Materials and Methods

### Electrolyzed water

**Fig 1:**
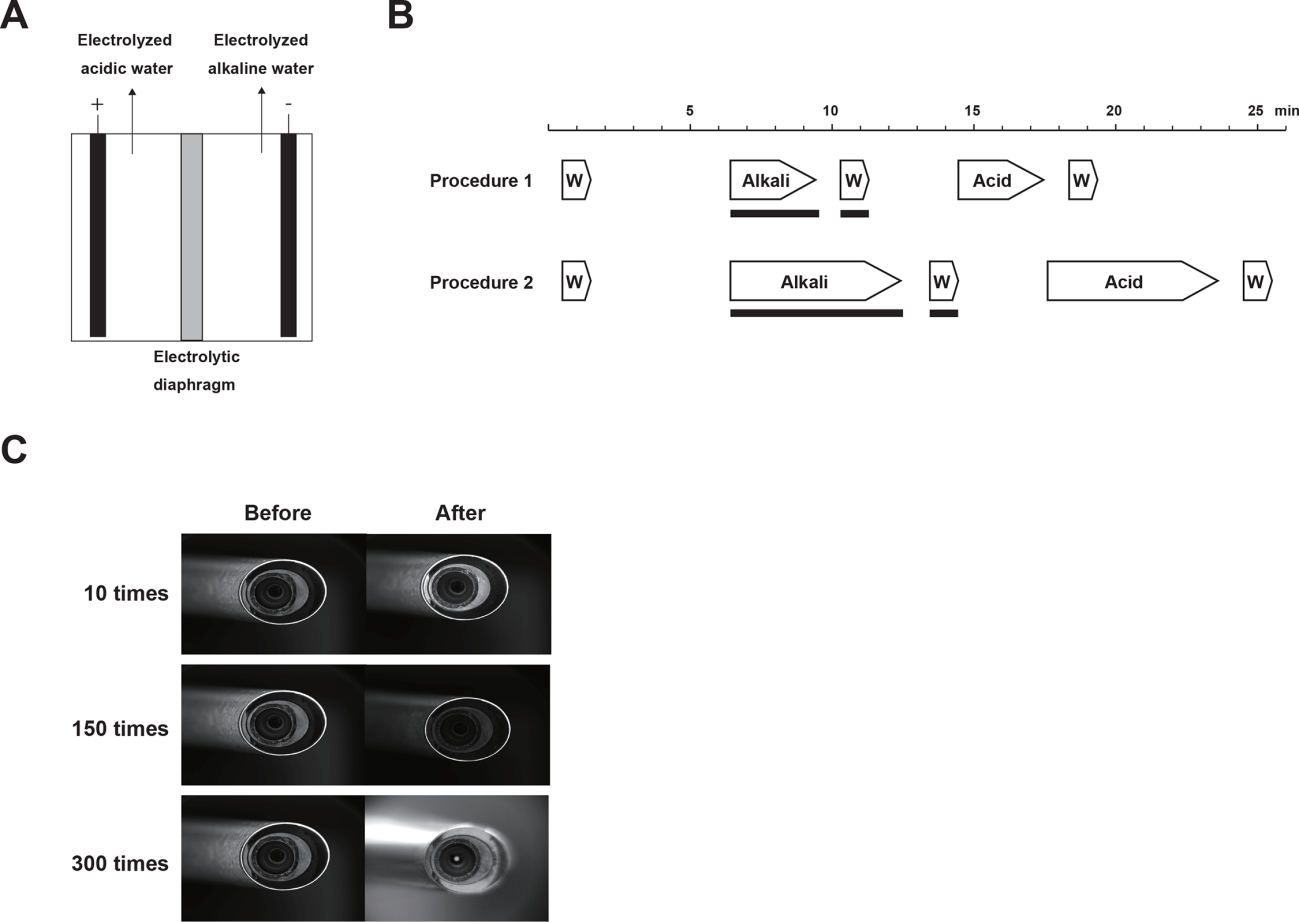
Electrolysis apparatus and washing procedures. (A) A schematic of the electrolysis apparatus: The apparatus consists of two wells separated by an electrolytic diaphragm. Anode and cathode plates were installed in the separate wells. Electrolyzed acidic and alkaline water were obtained from the anode and cathode wells, respectively. (B) Time schedules of washing procedures: Procedure 1 was used to determine the removal efficacy of bacteria, fungus and prions. Procedure 2 was used to evaluate the bacterial and fungal removal efficacy of procedure 1. Alkaline and acidic water steps were performed sequentially for 3 (Procedure 1), or 6 min (Procedure 2) each. The black box represents sonication. W: water. (C) Photographs of a rigid endoscope (502-457-030, Stryker, Kalamazoo, MI, USA) washed 10 (upper), 150 (middle) and 300 times (bottom) using procedure 1. The photographs are of the distal end of endoscopes before (left) and after washing (right).

Electrolyzed alkaline and acidic water were prepared in the electrolysis apparatus (Fig 1A). The apparatus consists of anode and cathode plates made of platinum-coated titanium separated by an electrolytic diaphragm (Y-9201T, Yuasa Membrane Systems Co. Ltd, Tokyo, Japan). Thirty-six liters of 0.15% NaCl solution was electrolyzed at room temperature at 27 V. Oxidation–reduction potential (ORP) and pH were measured using an electrometer (D-53S, Horiba Ltd, Kyoto, Japan) equipped with an ORP sensor (9300-10D, Horiba Ltd, Kyoto, Japan). Free chlorine content was measured using a chlorine meter (6560-10C, Horiba Ltd).

Our washing procedure consists of five treatment steps with water, alkaline or acidic water to perform disinfection. The duration of each step is indicated in Fig 1B. All processes were performed at room temperature. Stainless steel cylinders (SUS304; outer diameter 8.0 mm, length 10.0 mm) were purchased from Toyoshima Manufacturing Co., Ltd. (Osaka, Japan) and used as a model system for endoscopes. Cylinders were pre-washed in potable water for 1 min and then treated with electrolyzed alkaline water while being sonicated at 45 kHz for 3 min (Procedure 1) or for 6 min (Procedure 2). The cylinders were then rinsed in water with sonication for 1 min and treated with electrolyzed acidic water for 3 min (Procedure 1) or 6 min (Procedure 2), followed by a rinse with potable water for 1 min.

### Bacteria and fungus

*S*. *aureus* (ATCC 29213 strain) and *P*. *aeruginosa* (ATCC 27853 strain) were cultured in tryptic soy broth to 0.5 ± 0.2 McF units. Pre-cultured cells (15 mL) were incubated with stainless steel cylinders at 37°C for 24 h with agitation (250 rpm), and further incubated at rest at 37°C for 24 h. *C*. *albicans* (ATCC 60193 strain) was cultured in Sabouraud dextrose broth to 1.0 ± 0.4 McF units. Pre-cultured cells (15 mL) were incubated with stainless steel cylinders at 30°C for 48 h with agitation (250 rpm), and further incubated at rest at 30°C for 24 h. The cylinders were used to measure bioadhesion efficiency and the effectiveness of the washing procedures. Bacterial or fungal growth were assessed using the Association of Official Agricultural Chemists (AOAC) official methods [20, 21]. Sixty tests per strain were performed. Bacteria or fungus were collected from cylinders washed using our washing procedures (Procedure 1 or 2), from cylinders washed using unelectrolyzed water control (10 tests), or from unwashed cylinders, by sonication in 0.5 mL of physiological saline. Residual surviving bacteria were evaluated by the presence of colonies after spreading onto blood agar (Becton, Dickinson and Company (BD), Franklin Lakes, NJ, USA) for *S*. *aureus* or Mueller–Hinton agar (BD) for *P*. *aeruginosa*, and incubation at 37°C for 24 h (one agar plate per test cylinder). *C*. *albicans* was evaluated by spreading onto potato dextrose agar with chloramphenicol (Nikken Biomedical Laboratory, Kyoto, Japan) and incubation at 30°C for 24 h (one agar plate per test cylinder). The acceptable disinfection performance standard of the AOAC method is ≤ 2 positive plates for *S*. *aureus* and ≤ 3 positive plates for *P*. *aeruginosa* out of 60 tests. The number of residual bacteria on cylinders was indirectly estimated by counting the numbers of colony-forming units (cfu) on appropriate culture plates seeded with the effluent taken from the cylinders.

### Prions

Brain tissue from a human prion disease (sCJD) patient was obtained for use in this study. Written informed consent to participate in the study was given by the patient’s family. The protocol for our investigation was approved by the Ethics Committee of Nagasaki University Hospital (ID: 10042823), and the study was registered with the University Hospital Medical Information Network (ID: UMIN000003301). The protocol was also granted ethical approval for the use of brain tissue by the Japan Surveillance Unit for human prion diseases. A 10% (w/v) brain homogenate (BH) in phosphate-buffered saline (PBS) was prepared using a multi-beads shocker (Yasui Kikai, Osaka, Japan). After centrifugation at 2000 × *g* for 2 min, supernatants were collected and stored at −80°C. Stainless steel wires (SUS304, RKC Instrument Inc., Kanagawa, Japan; diameter 0.2 mm) were used as an endoscope model to determine washing efficiency for lowering prion infectivity. Wires were cut into 5-mm lengths, incubated with 2 μL of 10% BH, air-dried at room temperature for 1 day in a Petri dish, and then washed with procedure 1.

### Bioassay

Animal care and experimental procedures were performed in accordance with the Regulations and Guidelines for Animal Experimentation of Nagasaki University, reviewed by the Institutional Animal Care and Use Committee of Nagasaki University and approved by the president of Nagasaki University (ID: 1107040937). Mice were housed with access to food and water *ad libitum* in the Biomedical Research Center of Nagasaki University. All surgery was performed under isoflurane anesthesia, and all efforts were made to minimize suffering. Four-week-old human–mouse chimeric PrP knock-in mice, KI-ChM, which express PrP containing the sequence of human PrP between codons 23 and 188 and mouse PrP between codons 189 and 231, were used in this study [22]. Wires that had been incubated with 2 μL of 10% BH were washed ([Wire + BH + Wash]; N = 7) using the washing procedure 1 or unwashed ([Wire + BH]; N = 8), and were then individually implanted into the brains of anesthetized mice. Inoculations of 20 μL of 1% sCJD-BH into the brains of mice were used as positive controls ([PC]; N = 5). 20 μL of PBS ([NC]; N = 5) and implanted uncontaminated wires ([Wire]; N = 6)) were used as negative controls. We assessed the washing efficiency by monitoring the survival of inoculated mice. All mice were observed once a week for the appearance of transmissible spongiform encephalopathy-related clinical signs (waddling gait, rough coat, weight loss, very jumpy behavior, and hunched posture). Mice were observed daily at the onset of symptoms. Mice were sacrificed with the maximum attention to euthanasia and numbness using CO_2_ according to our guidelines. The brains were immediately frozen at −80°C.

### Western blotting

Brain tissue lysates were prepared by adding 10% BH to equal volume of lysis buffer (300 mM Tris-HCl, pH 7.5, containing 300 mM NaCl, 1% Triton X-100, 1% sodium deoxycholate and 4 mM EDTA). After 2 min of centrifugation at 10,000 × g, the supernatant was collected and its total protein concentration measured using the BCA protein assay kit (Nacalai Tesque, Kyoto, Japan). PrP^Sc^-protein was detected by adjusting the supernatant concentration to 2 mg/mL and digesting with 20 μg/mL of proteinase K (PK; Sigma Aldrich, St. Louis, MO, USA) at 37°C for 30 min. This was followed by boiling for 10 min with sample buffer (50 mM Tris-HCl, pH 6.8, containing 5% glycerol, 1.6% SDS, 100 mM dithiothreitol and a moderate amount of bromophenol blue). The proteins were separated by SDS-polyacrylamide (15%) gel electrophoresis and transferred onto a PVDF membrane (Immobilon-P; Merck Millipore, Damstadt, Germany). The membrane was blocked with a 5% solution of fat-free milk powder in TBST (10 mM Tris-HCl, pH 7.8, 100 mM NaCl, 0.1% Tween 20) and reacted with specific antibodies against PrP (SAF61) (SPI-BIO/Cayman Chemical, Ann Arbor, MI, USA) or β-actin (Sigma Aldrich, St. Louis, MO, USA). The membrane was further reacted with horseradish peroxidase-conjugated goat anti-mouse IgG antibody. Immunoreactive bands were visualized with an ECL prime western blotting system (GE Healthcare UK Ltd, Buckingham, UK).

### Statistical analysis

Survival data of mice were analyzed by the log-rank test using GraphPad Prism (GraphPad Prism Software, San Diego, CA, USA).

## Results

### Effect of the washing procedure on bacteria

The free chlorine concentration, pH and ORP of electrolyzed waters were measured at room temperature (Table 1). Both HCl and HOCl have the potential to damage stainless steel equipment. We washed three individual rigid endoscopes 300 times using procedure 1 and found no variation in surface appearance and image qualities of the three endoscopes (Fig 1C). We next examined the effectiveness of our washing procedure to remove bacteria *S*. *aureus* and *P*. *aeruginosa* or fungus *C*. *albicans*. We enumerated the bacterial or fungal binding efficiencies to the stainless steel cylinders and found *S*. *aureus*, *P*. *aeruginosa* and *C*. *albicans* were bound at 3.0 ± 2.5 × 10^7^, 4.9 ± 1.4 × 10^7^ and 6.9 ± 5.0 × 10^6^ cells, respectively (Table 2). Almost 10% of the cells in the cultures bound to the cylinders. We used these pre-treated cylinders to evaluate the bacterial disinfecting efficiency of our washing procedure. Procedure 1 insufficiently inhibited the growth of bacteria (Table 3). The number of positive plates were within the AOAC acceptance range of two or less for *S*. *aureus* and *C*. *albicans* but unacceptable for *P*. *aeruginosa*. However, the numbers of positive plates were within the accepted AOAC range for all three species tested using the longer wash times of procedure 2 (Table 3). When using procedure 1 without electrolyzed alkaline water, the number of plates positive for *S*. *aureus* and *P*. *aeruginosa* was above two. However, when using procedure 2 without electrolyzed alkaline water the number of positive plates was acceptable for all species. Inhibition of bacterial or fungal growth was not achieved using procedure 2 without electrolyzed water (unelectrolyzed water control), with all plates colonized (Table 4).

**Table 1:** Free-chlorine concentration, pH and oxidation reduction potential of electrolyzed water solutions.

	Free chlorine (ppm)	pH	ORP (mV)
**Unelectrolyzed**	2.33 ± 0.58	7.50 ± 0.08	780 ± 5
**Alkaline**	Not detected	11.91 ± 0.01	−804 ± 40
**Acidic**	18.67 ± 2.08	2.71 ± 0.08	1150 ± 3

ORP: oxidation-reduction potential. Values are the mean of triplicate results.

**Table 2:** Bacterial or fungal binding efficiency on metal surfaces (indirect measurement).

Strains	Bacteria attached (cfu/cylinder)	Concentration of bacteria immersion liquid (cfu/mL)
***S*. *aureus***	3.0 ± 2.5 x 10^7^	3.7 ± 2.1 x 10^8^
***P*. *aeruginosa***	4.9 ± 1.4 x 10^7^	4.8 ± 3.4 x 10^8^
***C*. *albicans***	6.9 ± 5.0 x 10^6^	4.4 ± 0.8 x 10^7^

**Table 3:** Number of cylinders still harboring culturable bacteria following treatments with electrolyzed alkaline and/or acidic water.

	*S*. *aureus*	*P*. *aeruginosa*	*C*. *albicans*
**Procedure 1**	2/60	6/60	0/60
**Procedure 2**	1/60	0/60	0/60
**Procedure 1 w/o Alkaline**	3/60	3/60	0/60
**Procedure 2 w/o Alkaline**	2/60	2/60	0/60

**Table 4:** Number of cylinders still harboring culturable bacteria following treatments with unelectrolyzed water control.

Strains	Positive decision of AOAC	After wash (cfu/cylinder)
***S*. *aureus***	10/10	4.0 ± 2.2 x 10^3^
***P*. *aeruginosa***	10/10	3.5 ± 1.0 x 10^3^
***C*. *albicans***	10/10	5.1 ± 6.2 x 10^2^

### Washing procedure effectiveness for human prions

**Fig 2:**
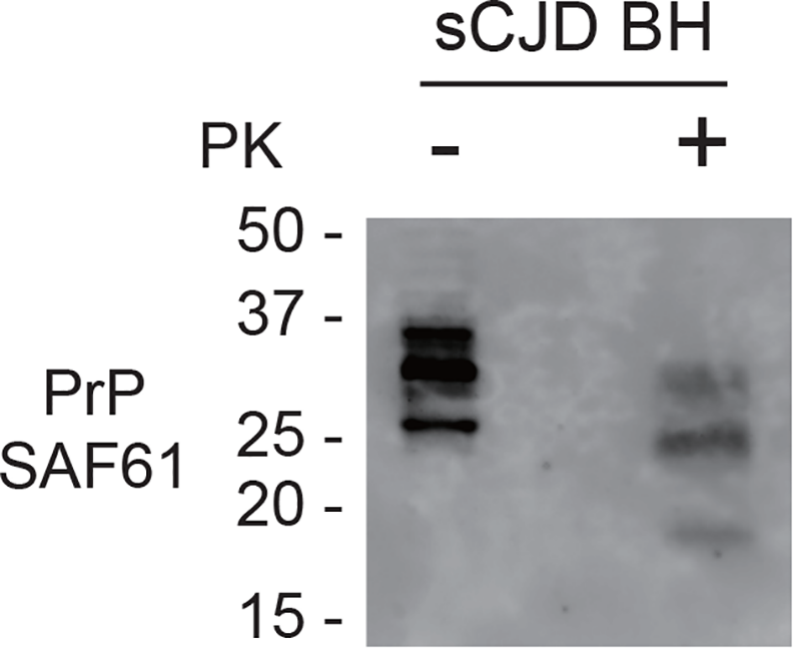
Existence of PrP^Sc^ in brain homogenates. PK-treated (PK+) and–untreated (PK-) brain homogenates from sCJD patient were loaded at concentrations of 40 and 20 μg of protein per lane onto a 15% polyacrylamide gel and subjected to SDS-PAGE. The proteins were detected by western blotting using anti-PrP antibody, SAF61.

**Fig 3:**
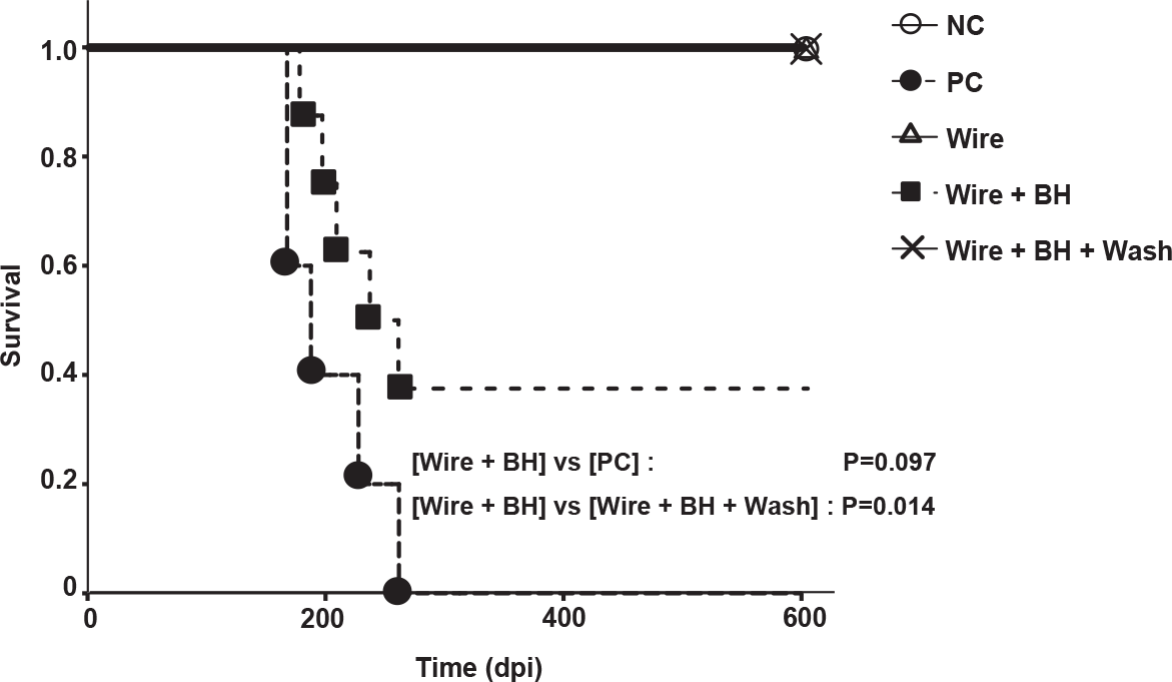
The new washing procedure can inactivate prions. Kaplan–Meier survival analysis of wire-implanted Ki-ChM mice. The statistical significances were evaluated using log-rank test.

**Fig 4:**
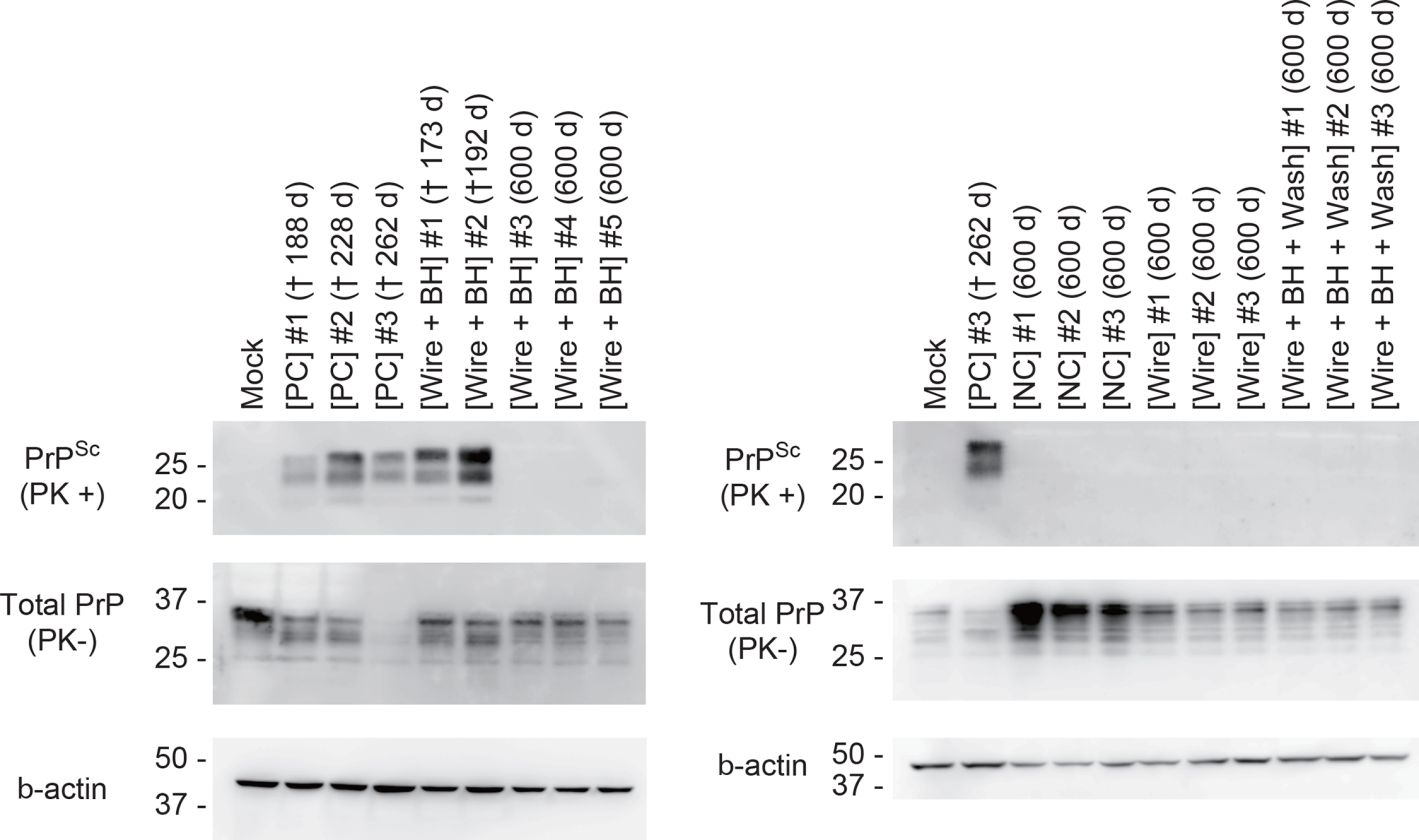
Evaluation of wash procedure efficacy by PrP^Sc^ detection in mice study. The proteins of PK-treated (PK+) and–untreated (PK-) brain homogenates from mice were detected by western blotting using anti-PrP (SAF61) and β-actin antibodies. The numbers above indicate the survival times or the dates when the mice were killed in days post-inoculation with prion or implants with wires.

Chimeric PrP knock–in mice (KI-ChM) expressing humanized chimeric PrP, including N-terminal human PrP followed by mouse PrP, were used to evaluate the decontamination efficacy of wires bearing the sCJD agent *in vivo* [22]. These mice are highly susceptible to sCJD [23]. The average incubation time for a prion dilution of 10^−1^ (2 × 10^−3^ g) has been reported as approximately 150 days [24]. The infectious titer of the BH was ~5.9 log of the LD50 per g. In this study, an individual wire was implanted into the brain of each KI-ChM mice. Western blot analysis demonstrated the existence of PrP^Sc^ in the starting sCJD material (Fig 2). All mice from the [PC] group developed prion disease (Fig 3), with a mean incubation period of 202.8 ± 41.2 days. A total of 62.5% (5/8) of mice implanted with [Wires + BH] developed prion disease, and the mean inoculation period in these five mice was 211.4 ± 32.9 days. There was no significant difference in the survival curve between the [PC] group and the infected [Wire + BH] group (log-rank test, P = 0.097). In contrast, all mice from the [Wire + BH + Wash] group survived and remained healthy during the observation period of 600 days after implantation (log-rank test between [Wire + BH] and [Wire + BH + Wash], P = 0.014). Western blot analysis showed that PrP^Sc^ were detected only in mice that developed prion disease and not in mice that remained apparently healthy (Fig 4).

## Discussion

In this study, we developed new washing procedures using electrolyzed water that are harmless to the human body, and evaluated their performance using several methods. The numbers of colonized plates from the washings of cylinders contaminated with bacteria and fungi were within the allowable range of AOAC official methods. Using washing procedure 2 without electrolyzed water decreased the bound bacteria and fungus on cylinders but was insufficient to completely remove pathogens (Table 4). Instead of direct staining of attached bacteria, residual surviving bacteria were collected from cylinders by sonication in 0.5 mL of physiological saline and cultured to quantify colony formation. Although we cannot exclude the possibility of incomplete recovery of bacteria from cylinder, we evaluated washing efficacy by counting the number of culture-positive cylinder. As expected, electrolyzed acidic water (pH 2.7) had a sufficient bactericidal effect alone, and prior washing with alkaline water did not enhance with this effect.

Heat-tolerant operative endoscopes and accessories should be sterilized using a vacuum-assisted steam sterilizer. However, this method is unsuitable for heat-sensitive endoscopes because of their complicated structures. Disinfection by immersion in 2% glutaraldehyde is sufficient to inactivate most bacteria and viruses including the hepatitis B virus [12]. However, glutaraldehyde is an irritant and sensitizing to the skin, eyes and respiratory tracts. A mild and safe method is needed for washing heat sensitive endoscopes [25].

The inability to easily detect prions on endoscopes and accessories remains a problem. In this study, we performed a bioassay using prion-contaminated stainless steel wires as a model system of surgical instruments to evaluate decontamination rates. Several reports have demonstrated that wires can bind prion seeds firmly, and that surface-bound prions can be transmitted by implantation of seeded wire into mice [26, 27]. Transmission experiments using KI-ChM mice were performed to evaluate the cleaning effect of our washing procedure for human prions. All treated wires were individually implanted into the brains of mice. Five of the eight mice implanted with wires inoculated with sCJD-BH developed symptoms. Although the reason for three mice remaining uninfected is unknown, it was estimated that about 10% of prions in the 10^−1^ BH adhered to a wire based on incubation time of the infected mice. Mice implanted with wires cleaned using our washing procedure did not develop symptoms of prion disease. Several reports suggest that prions must be decontaminated *via* a process of alkaline water treatment, such as 1 mg/mL of NaOH solution for 1 h [15, 16, 28]. This new, simple washing procedure, without the need for detergents or heating, will be useful to decontaminate equipment contaminated with pathogenic bacteria (*S*. *aureus* and *P*. *aeruginosa*), fungi (*C*. *albicans*) and prions. Further studies and tests using multiple and well-used instruments are needed before our procedure is recommended for clinical application.
